# On Origin and Evolution of the Antibody Molecule

**DOI:** 10.3390/biology10020140

**Published:** 2021-02-10

**Authors:** Umberto Oreste, Alessia Ametrano, Maria Rosaria Coscia

**Affiliations:** 1 Institute of Biochemistry and Cell Biology, National Research Council of Italy, Via P. Castellino, 111, 80131 Naples, Italy; umberto.oreste@ibbc.cnr.it (U.O.); alessia.ametrano@ibbc.cnr.it (A.A.); 2 Department of Environmental, Biological and Pharmaceutical Sciences and Technologies, University of Campania Luigi Vanvitelli, 81100 Caserta, Italy

**Keywords:** antibody, Ig domain, IgSF, evolution, immunoglobulin genes, immune system, somatic recombination, somatic hypermutation, RAG, Fc receptors

## Abstract

**Simple Summary:**

Like many other molecules playing vital functions in animals, the antibody molecule possesses a complex structure with distinctive features. The structure of the basic unit, i.e., the immunoglobulin domain of very ancient origin is substantially simple. However, high complexity resides in the types and numbers of the domains composing the whole molecule. The emergence of the antibody molecule during evolution overturned the effectiveness of the organisms’ defense system. The particular organization of the coding genes, the mechanisms generating antibody diversity, and the plasticity of the overall protein structure, attest to an extraordinary successful evolutionary history. Here, we attempt to trace, across the evolutionary scale, the very early origins of the most significant features characterizing the structure of the antibody molecule and of the molecular mechanisms underlying its major role in recognizing an almost unlimited number of pathogens.

**Abstract:**

The vertebrate immune system provides a powerful defense because of the ability to potentially recognize an unlimited number of pathogens. The antibody molecule, also termed immunoglobulin (Ig) is one of the major mediators of the immune response. It is built up from two types of Ig domains: the variable domain, which provides the capability to recognize and bind a potentially infinite range of foreign substances, and the constant domains, which exert the effector functions. In the last 20 years, advances in our understanding of the molecular mechanisms and structural features of antibody in mammals and in a variety of other organisms have uncovered the underlying principles and complexity of this fundamental molecule. One notable evolutionary topic is the origin and evolution of antibody. Many aspects have been clearly stated, but some others remain limited or obscure. By considering a wide range of prokaryotic and eukaryotic organisms through a literature survey about the topic, we have provided an integrated view of the emergence of antibodies in evolution and underlined the very ancient origins.

## 1. Introduction

Receptors that are capable of recognizing non-self molecular structures are found in a diverse array of multicellular organisms. Most of them belong to the immunoglobulin superfamily (IgSF) as containing the immunoglobulin domain. With the explosion of adaptive immunity, about 500 million years ago, the antibody molecule, also termed immunoglobulin (Ig), has become the key player of the immune defense system in jawed vertebrates. The basic unit consists of four polypeptide chains, of which two are defined as heavy (H) and two as light (L) chains [1]. The N-terminal end of each chain includes the variable (V) region responsible for the antigen-binding site, while the C-terminal portion of each chain represents the constant (C) region that mediates the antibody effector functions.

The structure of the gene locus encoding the H and L chains includes multiple copies of *V* (variable), *D* (diversity), *J* (joining) gene segments, which contribute to encoding the IgV domain through a mechanism of “somatic recombination.” These gene elements precede the exons encoding the C region. In the jawed vertebrates, two different arrangements of the Ig gene locus are known. In cartilaginous fish, multiple “clusters” of *V*, *D*, *J*, and *C* segments are present [2], whereas teleosts, similar to other vertebrates, are characterized by the so-called translocon arrangement consisting of multiple sets of *V*, *D*, and *J* gene segments, followed by a single copy of the exons coding for the constant domains. The IgV domain possesses a distinctive feature, that is a region, encoded by a *J* gene segment, which forms a diglycine bulge and is important for the V domain dimerization. These types of V domains are known as VJ sets or VJ V domains [3]. IgV and IgC1 domains derive from the very ancient Ig domain, which structurally looks like a compact parallelepiped of about 25 × 25 × 40 Å. It consists of 70–110 amino acids folded into a compact sandwich module formed by two facing β sheets of 7–9 antiparallel β strands that are connected by loops and stabilized by a conserved disulfide bridge covalently linking β strands B and F. Although varying significantly among jawed vertebrates, the amino acid sequence of the Ig domain throughout conserves two cysteines involved in the disulfide bridge, a tryptophan lying in the domain hydrophobic core and a leucine located in the center of the strand E [4]. A distinctive feature of the Ig domain is the ability to dimerize through its hydrophobic patch and form long chains by concatenating by its loops.

The main reason underlying the evolutionary success of the Ig domain certainly resides in its compact fold, which serves as the basic structure bearing the hypervariable loops involved in the formation of the antigen-recognition site. A key role in the folding dynamics is played by the clustering of the hydrophobic residues in strands B, C, E, and F. They are required for priming the folding nucleus of the Ig domain and for the formation of the disulfide bridge between strands B and F. In addition, to ensuring Ig domain compactness, this bridge in some way drives the formation of the Ig domain folding nucleus, thus preventing less stable or partially folded domains. The folding strategies that antibodies can adopt represent quality control checkpoints in Ig transport, ensuring a correct domain assembly [5].

In eukaryotes, four different types of Ig domains have been grouped in IgI, IgV, IgC1, and IgC2, differing in the presence or absence of additional β strands.

Here, we look through species that preceded the jawed vertebrates in an attempt to reconstruct the structural and functional properties that have resulted to be essential for the antibody assembly and to describe the most important steps that have led to its diversification in jawed vertebrates.

## 2. The Ig Fold in Bacteria

Immunoglobulin-like domains have been detected for a long time in prokaryotic surface proteins belonging to the intimin/invasin family, playing a role in host cell adhesion and pathogenicity [6]. Although very low sequence similarity (less than 20%), Ig-like domains of prokaryotes show a β-strand secondary structure similar to eukaryotic Ig fold.

Studies in Eubacteria and Archaebacteria [7] have raised the question of whether an Ig-like domain is present in distant phyla due to gene transmission or physicochemical properties that favor its structural assembly. Using hidden Markov modeling and BLASTP, matches between IgSF molecules and bacterial proteins strongly support the gene transmission view [8]. However, whether this transfer to different phyla of bacteria takes origin from a common ancestor, or results from independent bacteriophage-mediated phenomena of horizontal gene transfer remains an open question.

Given objective difficulties in identifying which prokaryotic Ig fold is more closely related to the eukaryotic Ig domain, the prokaryotic one has been classified as bacterial Ig domain (BIg). Bateman et al. [8] underlined similarities of BIg to the eukaryotic IgI domain, proposing that it can be considered the most ancient conformation from which the four eukaryotic basic sets are derived. However, there is no clear evidence supporting this topic.

**Figure 1:**
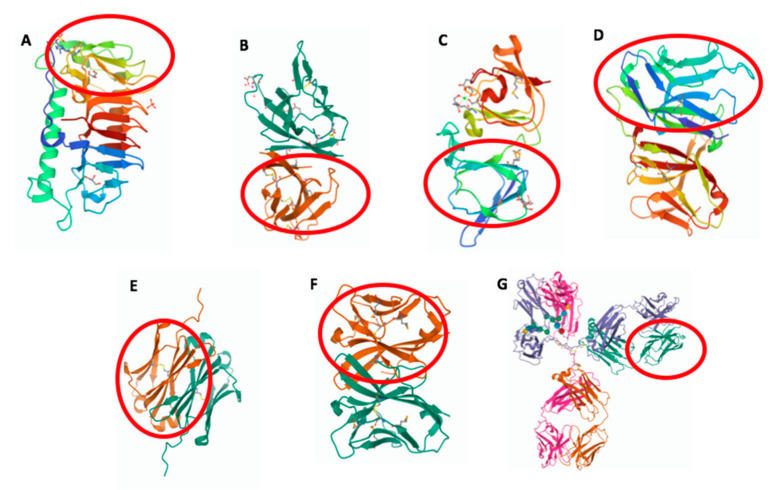
Three-dimensional structure of molecules from different species containing an IgV-like or IgV domain (in red circle). (**A**) *Shewanella frigidimarina* ice-binding protein_1, PDB entry: 6BG8; (**B**) SARS-CoV-2 ORF8, PDB entry: 7JX6; (**C**) *Geodia cydonium* geodin, PDB entry: 4IAU; (**D**) *Branchiostoma floridae* VCBP, PDB entry: FBO; (**E**) *Ginglymostoma cirratum* IgNAR V domain, PDB entry: 2I27; (**F**) *Ictalurus punctatus* NITR11, PDB entry: 2QQQ; and (**G**) *Mus musculus* IgG1, PDB entry: 1IGY.

Structural analyses of BIg domains from various bacterial species reveal unique traits of these domains with respect to the eukaryotic Ig domain. A Ca^2+^ binding module has been identified in BIg domains of *Leptospira* [9] and *Strepococcus pneumonia* [10] proteins. Similar to the canonical Ig domains, the Ca^2+^ binding site has a secondary structure composed of β strands, but it shows low sequence homology and is distinguished by the absence of the core conserved disulfide bridge. BIg domains are frequently found in surface proteins as important structural and functional elements. A number of explanatory examples are given below. A component of the fimbrial structure of *Streptococcus suis* contains BIg domains that promote adherence to host cells [11]. BIg domains of the *Porphyromonas gingivalis* T9SS protein cover a role in the secretion of virulence factors [12]. *Salmonella* BapA protein, which is important for biofilm formation, comprises tandem BIg repeats [13]. The flagellin protein contains BIg domains that have also a well-recognized role in flagellar motility [7]. BIg containing proteins of plasmid incompatibility, expressing multiple determinants for antibiotic resistance, are required for plasmid conjunction [14]. The Antarctic bacterium *Shewanella frigidimarina* possesses ice-binding, protein-containing BIg domains that allow adsorbing their host bacterium onto ice [15] (Figure 1A). Many of the above-mentioned functions imply a homo- or hetero- recognition mechanism, indicating that a functional specificity remains highly conserved among species.

## 3. The Ig Fold in Viruses

During co-evolution with their hosts, viruses frequently gain genes by horizontal transfer, and change or multiplying them, providing a source of new functionalities with the potential to hinder or simulate the previous host functions [16]. Interestingly, a number of host-derived viral genes are mainly involved in immune response, encoding molecules of the innate immune system belonging to the IgSF, such as those involved in lymphocyte activation or signaling. On the contrary, no homologs of the IgSF adaptive immune molecules have been found.

An extensive survey of genome databases provides evidence that different viruses independently acquired the Ig domain. Seventeen distinct viral Ig domain families have been defined in a wide range of animal viruses on the basis of new Ig domains, identified by multiple-sequence based protein models [17]. Although these viral Ig domains are highly divergent, most of them share a similar architecture, consisting of an N-terminal signal peptide, one or more Ig domains, and a C-terminal transmembrane region. However, the reason for their widespread distribution in viruses is most probably related to their functional role rather than to structural features.

Because of its conformational plasticity, the acquired Ig domain is particularly advantageous for viruses in terms of providing remarkable functional diversity, e.g., mediating cell adhesion [18]. An explanatory example is the CR1 Ig domain protein from adenoviruses that impairs the surface expression of class I major histocompatibility complex molecules by inhibiting their transport pathways in host cells [19].

Another example comes from the glycoprotein E2 of the hepatitis virus C, which contains the Ig-like domain that is essential for virus entry into the host cell. The conformational variability of the Ig-like domain allows some of the hydrophobic core residues to be exposed on the viral surface, promoting changes in the secondary structure of one β strand in such a manner that one viral particle can simultaneously display on its surface E2 molecules in different folded shapes [20].

Novel families of Ig domain proteins have been found also in α- and β-coronavirus, in which they can modulate the host defense system by inhibiting or delaying the immune response against viral invasion [17]. The ORF8 protein of SARS-Cov-2 is known as one of the so-called accessory proteins; it does not have a primary role in viral replication but, similarly to the adenovirus CR1 proteins, might have a pathogenic potential to delay the host immune response [17]. ORF8 and several other coronavirus proteins comprise Ig-like domains (Figure 1B), which share general structural features with other Ig domain proteins from animal viruses, such as the typical pattern of two cysteines forming the canonical disulfide bridges. However, the Ig domain is one of several rapidly evolving regions identified in the SARS-CoV-2 genome and might have mutated during cross-species transmission.

Ig-like domains have been identified also in surface proteins of double-stranded DNA bacteriophages [21], indicating that horizontal gene transfer has frequently occurred between viruses and bacteria. This is also emphasized by the structural similarities between viral and BIg domains.

At present, the function of the bacteriophage molecules carrying Ig-like domains is still obscure. However, there is some evidence that, during the phage infection, Ig-like domains can interact with bacterial cell surface carbohydrates, facilitating phage adsorption. The presence of an Ig domain in viruses and its ability to interact with host cells clearly place this domain at the center of the immune offense-defense network.

## 4. The Eukaryotic IgV Domain

**Figure 2:**
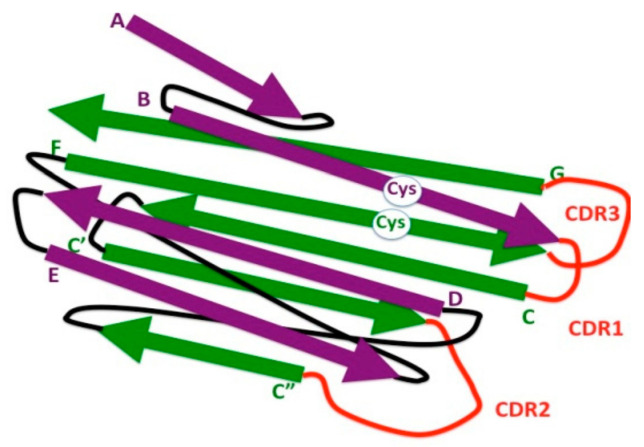
Schematic representation of 3-D strand topology in the eukaryotic IgV domain. Antiparallel strands of the two β sheets are colored in purple and green, respectively, and identified by letters. Connecting loops are depicted in black. Loops bearing the complementary determining regions (CDR1, CDR2, and CDR3) are depicted in red. The highly conserved cysteine residues (Cys) involved in the formation of the disulfide bond between the β strands B and F are indicated (white circle).

The N-terminal ends of IgL and IgH chains consist of IgV domains that associate with each other to form a single antigen-binding site. IgV can be distinguished from IgC domains by the high sequence variability required for antigen recognition and by the presence of two extra strands (C′ and C″) located in the middle of the domain (Figure 2).

Another distinct feature of IgV domains is the presence of a 65–75-aa long stretch, between the two highly conserved cysteines involved in the formation of the disulfide bridge. Instead, the corresponding region in the IgC domain comprises 55–60 residues [22]; it can be even shorter in some Ig-like domains [23]. A high degree of sequence variability resides in the loops connecting B to C, C′ to C″, and F to G β strands, called complementary determining regions (CDRs)—CDR1, CDR2, and CDR3, respectively. It should be noted that the β strand G is not encoded by the same gene segment (*V*) as that encoding the rest of the domain, but by a different gene segment (*J*).

It has been argued that the Ig domain originated by duplication and divergence from an ancestral domain. A half-domain prototype has been hypothesized to consist of the β-strand ABCC’ or GFED, which is associated in a homodimeric form in the same manner as these β strands associate in the IgV domain. The presence of introns in the middle part of the sequence encoding the region between the conserved disulfide bridges is in line with the hypothesis of a half-domain origin [24]. In addition, the discovery in ancient invertebrates of homologs of Thy-l, a thymocyte cell surface protein with a single IgV domain [25], has fueled the concept that the IgV-like fold is the most ancient type.

Interestingly, a gene encoding an adhesion molecule (GSAM) that includes an Ig domain with high sequence similarity to the IgV domain of vertebrate L chains, has been identified in a living fossil, the marine sponge *Geodia cydonium* (Porifera) [26] (Figure 1C). Notably, the presence of CDR1-like regions within the GSAM Ig-like domain and the identification of signs of mutational events represent remarkable clues of the primordial steps toward the emergence of the mechanisms generating antibody diversity [26]. Immunological studies conducted in protochordates turned out to be particularly useful for better understanding the origins of the IgV region as the basic structural module committed to immune recognition. Protochordate amphioxus has been positioned as the basal group of the phylum Chordata and it represents the sister group of the tunicate—vertebrate clade [27]. Although lacking the classical adaptive immunity, amphioxus presents an unexpected complexity and diversity of the innate immune response [28]. Thus, this species represents a remarkable reference for the comparative studies on the evolution of the vertebrate immune system. The amphioxus *Branchiostoma floridae* [29] and the urochordate *Ciona intestinalis* [30] have been shown to possess variable region-containing chitin-binding proteins (VCBPs), which are immune recognition molecules comprising two tandem IgV domains and a single chitin-binding domain (Figure 1D). An in-depth analysis of the VCBP structure revealed that the hypervariable sites are localized on the β-sheet surfaces of the IgV domains rather than on the connecting loops, as found in the Igs of jawed vertebrates. VCBPs lack a characteristic contiguous *J*-like segment but show a particular folding in the strand G, which is a key constraint on the dimerization of IgV regions. The high-resolution structure of VCBP3 revealed that IgV domains are packed in a head-to-tail fashion, in contrast to all jawed vertebrate IgVs, found to pack in a head-to-head configuration [31]. The presence of IgV domains ensures the binding of VCBPs to gram-positive and gram-negative bacteria, promoting hemocyte phagocytosis of pathogens [32]. This opsonizing activity is reminiscent of that typically observed for vertebrate antibodies, illustrating an evolutionary origin of this function. Intriguingly, an Intermediate-type Ig domain has been identified at the C-terminus of a putative C-type lectin from the ascidian *Botryllus schlosseri*. This tunicate lectin is the first example of a soluble protein that uses also an Ig domain for recognition, in addition to the canonical carbohydrate-binding domain [33].

In AmpIgVJ-C2, another molecule from *B. floridae*, an IgV domain containing a *J* segment-like structure has been described [34]. The presence of the typical β-bulge in the strand G provides evidence that AmpIgVJ-C2 belongs to the same family as the antibody molecules. This conclusion is also supported by crystallographic data obtained for recombinant molecules. Interestingly, the head-to-head arrangement of the IgV domains and their packing mode observed in the dimer are common features shared by AmpIgVJ-C2 and antibodies. It is worthy of note that AmpIgVJ-C2 is the only IgV domain homodimeric structure found in invertebrates, reminiscent of the L chain homodimer of the human Bence-Jones protein [35].

At present, we can conclude that important features of the antibody V domain can be traced back to early chordates, before the appearance of the IgC1 domain.

Aside from Ig, the IgV domain is also present in several molecules of the innate immune system of jawed vertebrates. Of particular interest is the case of the novel immune-type receptors (NITRs), found in teleost fish [36,37]. NITR genes encode type I transmembrane receptors, which contain one or two extracellular Ig domains (Figure 1F) and resemble molecules that serve as activating or inhibitory receptors. Since lacking the basic mechanisms for somatic diversification but possessing a *VJ* segment continuity, such non-rearranging Ig-like genes would presumably have been served as primordial substrates that have led to the emergence of recombining antigen-binding receptors [38,39]. Evolutionary and comparative studies indicate that NITRs are likely to have diverged from an Ig gene cluster following a gene birth-and-death process, which accounts for their rapid diversification [40], a feature in common with Igs and killer-cell immunoglobulin-like receptors (KIRs) [41]. Apart from Igs, NITRs may represent the sole example of a receptor gene complex encoding a diversified family of V regions in vertebrates and represent a compelling case for parallel evolution of V regions of both adaptive and innate immune receptors [42].

Looking across the tree of life over evolutionary timescales, we have accumulated in the previous paragraphs some clues about the non-vertebrate origin of the ancestral building blocks of the antibody molecule. Here, we have underlined that the IgV domain has been widely used by the recognition molecules of earlier vertebrates. However, only in jawed vertebrates, the V domains have acquired a structural significance to form the genuine antigen-binding site, along with the potential to vary in order to cope with a multitude of different antigens.

## 5. Somatic Recombination

Somatic recombination is a complex genetic mechanism occurring during the different stages of B cell maturation. It consists of a random rearrangement of just one gene segment of each *V*/*D/J* set to encode a complete V domain. In the *H* chain gene locus, it occurs in two steps, namely, *D–J* in pro-B cells and *V–DJ* in pre-B cells; in the L chain gene loci, it occurs in pre-B cells. The complex of the two endonucleases, encoded by the recombination activating genes *RAG1* and *RAG2*, cut the DNA sequence of *H* and *L* chain gene loci at specific positions defined as recombination signal sequences (RSSs). RSSs are adjacent to *V*, *D*, and *J* gene segments and consist of conserved heptameric and nonameric sequences separated by 12- or 23-bp long spacers. *RAG1* encodes a 1043-aa homodimeric protein, which carries the ability to recognize RSSs and cleave DNA; *RAG2*, encoding a 527-aa protein, cooperates in the recombinase activity together with other accessory factors. The recombination enzymatic complex induces the juxtaposition of the RSSs flanking the *V*, *D,* or *J* segments, cuts the DNA, and produces a circular fragment, consisting of the region between the two gene segments, and two truncated coding ends, which are ligated by the Artemis multi-enzymatic complex, including a kinase and the Terminal deoxynucleotidyl Transferase (TdT). The latter adds extra nucleotides to the rearranging junction.

The mechanism of the *VDJ* recombination has been found only in jawed vertebrates and it is specific for Ig and T-cell receptor (TCR) gene loci. However, precursors of the components of the *VDJ* combinatorial machinery have been identified in invertebrate species.

In the amphioxus *B. floridae* the *BfRAG1L* gene has been shown to encode the functional central domain of vertebrate *RAG1*. Recombinant *BfRAG1L* contains a site motif of retroviral type II nuclease, able to cleave RNA and DNA. Moreover, it recognizes vertebrate RSS in presence of mouse RAG2 [43]. These results support the idea that one or more proteins, probably involved in viral recombination, have been transposed in the vertebrate genome, where they acquired the function of mediating the *VDJ* recombination process [38].

*RAG1*- and *RAG2*-like sequences have been identified in different invertebrates, e.g., the sea urchins *Lytechinus variegatus* and *Patiria minata*, and the mollusk *Aplysia californica*. *Strongylocentratus purpuratus SpRAG1L* and *SpRAG2L* genes show a genomic organization similar to that of vertebrates [44]. They are transcribed and spliced during embryogenesis, and the encoded proteins assume a conformation such that the residues that are essential for the activity in vertebrate homologs become exposed. This suggests that vertebrate *RAG* genes were already present in the deuterostome ancestor and that their role in adaptive immunity has been a recent acquisition.

In addition, the Artemis nuclease, which participates in the recombination process to cleave the DNA hairpin extremities generated by the double-stranded breaks, possesses an ancient origin, as suggested by the identification of Artemis-like sequences in fungal and metazoan species [45].

It seems reasonable to hypothesize that two evolutionary lines have driven precursors of recombination enzymes and immune receptors to meet each other at a given stage of jawed vertebrate evolution, providing the bases for the adaptive immunity.

## 6. Somatic HyperMutation

In order to fight an almost unlimited number of pathogens, the adaptive immune system, in addition to the recombination mechanism for the generation of the primary antibody repertoire, relies on another process, the somatic hypermutation (SHM), which drives further diversification of the antibody variable regions by generating higher affinity variants (the so-called affinity maturation).

The 24-kDa enzyme activation-induced cytidine deaminase (AID) initiates SHM by deaminating cytosine to uracil in the context of the so-called mutational hotspot motif RGYW (R = A or G, Y = C or T, and W = A or T) in single-stranded DNA. Thus, the C:G pair is converted to U:G mismatch; this is repaired by base excision repair enzymes, and error-prone DNA polymerase fills the gap creating mutations as well insertion/deletions. AID is a member of the APO lipoprotein B mRNA editing enzyme catalytic polypeptide-like (APOBEC) family, which edits messengers, also described as DNA mutators.

After discovery by Tasuku Honjo and Michael Neuberger in 1999 [46,47,48], recent work has identified several largely diversified *AID/APOBEC* genes across metazoans, underlining their involvement in several biological processes, with a primary role in immune defense [49]. Identification of AID/APOBEC deaminase family members in invertebrates may contribute to understanding the evolutionary split of innate and adaptive immunity. Modifications of exogenous genetic material is a well-known process, adopted by bacteria as a defense strategy against viral infections, by using restriction enzymes and CRISPR/Cas9 systems [50]. Gene expression analyses in different invertebrate phyla led to the identification of a family of genes encoding AID/APOBEC homologs [51], indicating a very ancient immunological role. The ancestral AID/APOBEC family members are derived from a branch of the zinc-dependent deaminase superfamily in the early stage of vertebrate radiation [52]. AID homologs capable of triggering SHM have been identified in bony fish [53] and cartilaginous fish [54], which both have Ig genes, but also in a jawless vertebrate, the lamprey [55], which does not. The presence of an AID homolog in the lamprey is remarkable, as its system of adaptive immunity is not based on antibodies, but on variable lymphocyte receptors (VLRs) [49].

To date, the best molecular evidence for affinity maturation in early gnathostomes comes up from the discovery of an Ig isotype exclusive to nurse sharks, that is IgNAR. The very limited number of IgNAR *V*-elements shed light on the origin of Ig somatic hypermutation, dating it back before the radiation of bony fishes. After hyperimmunizing a nurse shark with hen egg lysozyme, *IgNAR* genes encoding specific antibodies with increased binding affinity were isolated, reinforcing the existence of an affinity maturation process [56]. Singularly, it has been reported that IgNARs possess distinct mutational patterns compared to those found in teleost fishes [57,58]. It can be concluded that the mutability of the immune receptors is widely spread, although the basic mechanisms involved may vary among different species.

Moreover, investigations on the diversity of the V domains of IgM H chains from *Trematomus bernacchii*, an Antarctic teleost species, have uncovered several peculiar features reminiscent of the mammalian hypermutation mechanisms. A high mutation rate in hotspot motifs, high accumulation of nucleotide substitutions in CDRs, a bias for transitions over transversions, and an unusual excess of insertions and deletions have been observed. These findings provide an example of higher CDR mutability in a cold-adapted fish than that observed in other actinopterygian species [59,60].

## 7. The Ig Domain in Adaptive Immune Receptors: IgC1

**Figure 3:**
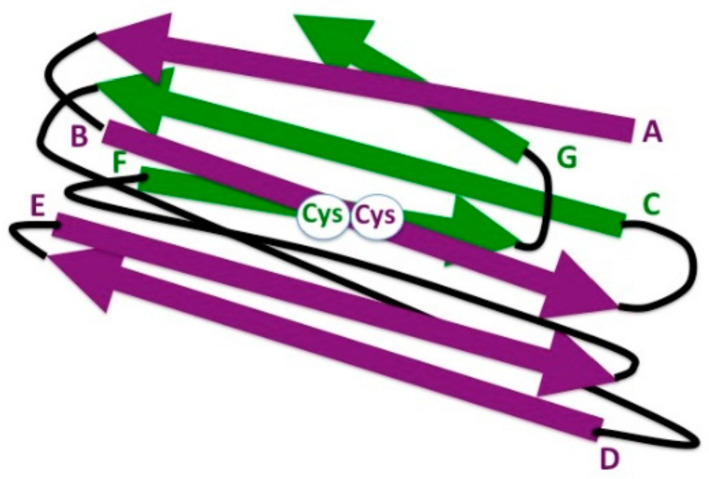
Schematic representation of 3-D strand topology in the eukaryotic IgC1 domain. Antiparallel strands of the two β sheets are colored in purple and green, respectively, and identified by letters. Connecting loops are depicted in black. The highly conserved cysteine residues (Cys) involved in the formation of the disulfide bond between the β strands B and F are indicated (white circle).

The IgC domain comprises two β sheets, one formed by β strands A, B, E, and D, and the other one by strands G, F, and C, which are packed face to face with an angle of 30°; a disulfide bridge links strands B and F. There are two types of IgC domains—IgC1 (Figure 3) and IgC2. IgC1 can show, in some cases, the additional very short strand C′; IgC2 lacks the strand D but has a strand C′ similar to that of the IgV domain [61].

Very interestingly, while the IgC2 domain is present in numerous molecules in both vertebrate and invertebrate animals, such as lectins, cell adhesion molecules, connectis, major sperm, and zona pellucida proteins, the IgC1 domain is limited to jawed vertebrates and has been found only in the adaptive immune molecules, e.g., immunoglobulin, TCR, Major Histocompatibility Complex class I and II, and CD1. This observation supports the idea of the contemporary emergence of all the actors playing a major role in the adaptive immune response during the so-called immunological big bang, all using the novel IgC1 domain type [62].

Since all these molecules possess at least a pair of IgC1 domains of different polypeptide chains facing each other, we launch the idea that this specific ability to dimerize might account for the evolutionary novelty introduced by the IgC1 domain. In contrast, IgC2 domains have not been found in a dimeric form in invertebrates. In this regard, the dimeric structure was confirmed for the IgV domain of the amphioxus AmpIgVJ-C2, but not for the IgC2 domain [34]. In our view, the dimeric structure of all these receptors appears to be crucial for the signal transduction of their membrane-bound forms upon the occupancy of the antigen-binding site. Since conformational transitions of both H and L chains are related to the bound or unbound state, the evolutionary shift from IgC2 to IgC1 has thus unearthed a suitable feature of IgC1 for the adaptive immune response. However, it is very difficult to assess what characteristics of the IgC1 domain permit dimerization. Generally (but not always), in the dimeric structures, the facing beta-sheets are those containing β strands G, F, and C, generally (but not always) including a short strand C’. Based on the analyses of a data set of 47 immunoglobulin molecular structures, the contact surface between IgC1-IgC1 domain pairs of 630 Å has been measured; 12 residues, which are half of those involved in the pairing, are conserved in hydropathic character, sequence, and position. They are in a close relationship with the residues involved in the Ig fold [63].

## 8. Evolution of the Antibody Architecture

### 8.1. The Modular Structure

An antibody is a complex molecule consisting of four polypeptides, two H and two L chains—the two H chains are linked to each other by disulfide bridges, and each L chain is covalently linked to an H chain, even though exceptions occur. For instance, the human IgA2m1 allotype has been reported to carry a disulfide bond between the constant regions of the two L chains, instead of the canonical H–L linkage [64]. The IgV domain is located at the amino-terminus of an L chain and is followed by a single IgC1 domain. The IgC1 domains of the H chain are assembled in head-to-tail strings so that the loops connecting β strands B to C and D to E in the preceding domain face those connecting A to B and C to D of the next domain; only the penultimate domain of the H chain shows a 180° rotation around the longitudinal axis so that the two domains are in a parallel configuration and their orientation is stabilized by glycans linked to a conserved asparagine residue. The C-terminal domain pairing of the two H chains is different from that described above because it is more compact and hence stabilizes the entire structure of the antibody molecule. The last domain is followed by a short tail, called the secretory tail.

The number of C1 domains in the Ig H chain is particularly large in fish, ranging from 2 (*Takifugu rubripes* IgH) [65] to 15 domains (*Danio rerio* IgD) [66]. Cartilaginous fish are the oldest vertebrates shown to have an antibody-based adaptive immune system. They have radiated from a common ancestor of all jawed vertebrates about 450 million years ago and hence, given the key position they occupy in evolution, studies performed on their antibodies are particularly informative. In fact, there exist many differences between the immune system of cartilaginous fish and that of other vertebrates. As already mentioned above, the structure of the *H* chain gene locus in cartilaginous fish consists of multiple mini-clusters, each composed of one *V*, two or three *D*, and one *J*, followed by one set of *C* region exons; some clusters are unique because carrying partially (*VD* or *VJ*) or completely (*VDJ*) recombined germline gene segments. A varying number of clusters (up to 100) has been found in different species [2].

Three different H chain isotypes are present in these fishes—IgM, IgW (previously named IgNARC, IgX, or IgR) orthologous to IgD, which is present in all jawed vertebrates, and IgNAR [67]. IgNAR exists in two forms differing in the number of IgC1 domains (three or five). It has been suggested to be the most ancient antibody isotype [68] and represents a peculiar case that appeared in the evolutionary history of antibodies, being a functional antibody devoid of L chains; this unusual feature has been also found in camel H-chain-only antibodies (HCAbs), which lack the first constant domain (CH1) in which resides the cysteine responsible for binding to the L chain [69]. The antigen-binding site of these antibodies is thus composed of a single V domain referred to as VHH. This similar trait occurring in distantly related species is an example of convergent evolution.

We can try to formulate some hypotheses to explain the existence of these naturally occurring antibodies devoid of the L chain, despite its encoding gene is present and functional, as demonstrated by the presence of L chains associated with H chains in the other two isotypes. In IgNAR, the CH1-CH1 homodimer has been shown to have a dissociation constant Kd of about 600 nM suggesting a stronger interaction than that found for other CH dimers, e.g., CH3-CH3 dimers show a Kd of 188 μM [68]. As a consequence, the interaction strength limits the CH1-VH flexibility and guides VHs orientation by 180° apart from each other.

Moreover, the absence of L chains could be due to a different mechanism adopted for assembly in cartilaginous fish. The release of newly synthesized H chains from the endoplasmic reticulum may not depend on the encounter with the chaperone BiP, as it happens in teleosts and other vertebrates. Generally, the CH1 domain does not automatically fold during synthesis but, to properly fold, it first needs to encounter the BiP peptide and subsequently associates with the L chain. BiP and IgL chains cooperate to control the folding of the H chain and ensure the fidelity of antibody assembly [69,70].

The shark IgNARs display another distinct feature compared to conventional antibody architecture. The V domain of IgNAR comprises a particularly long and structurally complex CDR3 loop, which ensures a higher sequence diversity of the antigen-binding site, compensating for the absence of VL domains (Figure 1E). Furthermore, extra disulfide bridges stabilize the V domain and, at the same time, position the extended CDR3 loop in such a way to allow access to antigen [42,71]. Structural studies have unveiled that the antigen-binding sites of IgNARs show a binding interface and nanomolar affinity comparable to those of conventional antibodies, despite they contain only two of the three CDRs. Interestingly, the conformational plasticity of the CDR3 loop has occurred several times during evolution as an alternative mode to broaden the antibody diversity when the repertoire of V domains is very limited.

An additional isotype, named IgT, has joined the IgM and IgD with the emergence of bony fish, but it is absent in tetrapods [72]. It has been ascertained that this isotype performs its functions mainly within the mucosal compartments [73], thus it can be considered orthologous to amphibian IgX and to IgA of mammals. In trout IgT, the CDR3 is longer (5–10 aa) than that of IgM VH (4–5 aa). This finding suggests that the more extended CDR3 in IgT might serve to recognize a wider range of pathogenic epitopes [74]. The interesting question raised by Zhang et al. [75] of whether the IgT basic unit H_2_L_2_ dimerizes, thus resembling mammalian IgA, or polymerizes as tetramers such as teleost IgM, or exists as a monomer such as IgG, outlines the extraordinary intrinsic plasticity of the antibody architecture, permitting to exist as monomers or with different degrees of polymerization.

### 8.2. The Dynamic Structure

A fundamental role in the evolution of the antibody architecture is played by the so-called hinge region—the key region that accounts for the functionality of the antibody, which is located in the middle of the molecule. Hinge presents a highly variable amino acid composition, particularly rich in cysteines and prolines—cysteines are used to bridge the two H chains; prolines confer conformational flexibility. Moreover, the length of the hinge greatly influences Fab–Fab flexibility [71].

A flexible hinge allows (i) functional motions of Fab arms, the portions of the molecule containing the two L chains, and the two N-terminal domains of the H chain (IgV followed by the first constant domain) favoring the binding of antigens of various shapes and sizes, and (ii) triggers the effector activities performed by the region called fragment crystallizable (Fc), which consists of the remaining part of the molecule with the exception of the Fab arms [76].

During vertebrate evolution, the hinge region first appeared in teleosts. A hinge region, encoded within the CH2 exon, has been highlighted in *T. rubripes* IgH chain [65]. This hinge has several distinct features such as the presence of a VKPT stretch repeated five times but lacks the cysteine residue to form a disulfide bridge between the two H chains.

Once acquired, the structural complexity of the hinge increased during evolution, along with antibody configuration. As a result, mammals exhibit the most diversified hinges within tetrapod species [77].

The first example in the evolution of a hinge encoded by a distinct exon has been found in the amphibian IgF isotype [78]. The hinge region identified in the *Xenopus tropicalis* IgF forms a loop connecting the CH1 and CH2 domains, possibly acting as a flexible spacer. The presence of a conserved cysteine, likely involved in a covalent bridge between the two IgF H chains, and of three prolines recall the characteristic features of the mammalian Ig hinge regions.

Placental mammals (Eutheria), including humans, possess five IgH chain isotypes— IgD, IgA, and the IgG; all consist of three CH domains and a hinge region that joins the Fab arms to the Fc region [79]. Conversely, IgM and IgE H chains have an additional CH domain in place of the hinge region.

Over the past decades, different theories regarding the origins of the hinge have been formulated. One theory proposed that the hinge is encoded by an exon that evolved in parallel with the immunoglobulin genes [80,81], and it might be related to the V region, according to the findings derived from non-human primates [82]. Others suggested that the hinge region exon might have evolved by mutation of the CH2 domain-encoding exon [83], as supported by the presence of the C-terminal cysteine and proline residues of the bird IgY CH2 domain observed at the C-terminus of many mammalian hinge regions [84,85]. Another option is that it may have evolved from gene duplication of a small constant region [80], or from contraction or extension presumably of the CH2 domain [86]. A similar case has been found for Antarctic fish IgM, which display a remarkably long hinge region (15–24 aa) derived from an elongation of the CH2 exon; it is expressed in both the secretory and membrane forms [87,88]. Another striking case comes from IgT, which has been found in Antarctic fish to lack the most second constant domain. The remnant domain retained just a few amino acids (8–17 aa), mainly those typical of hinges, e.g., prolines and cysteines [89]. Antarctic fish Igs are witnesses of evolutionary genomic changes whose products might have been preserved since appropriate for the structural flexibility. Hence, the hinge can be considered a structural linker between the Ig V and C domains and a critical driver to guarantee all the appropriate movements for exerting antibody function.

We will continue the long evolutionary history of the antibody molecule, covering another important building block, the L chain, although detailed knowledge of the evolutionary history of *IgL* genes across species remains elusive. Diversity has a major role in the antibody repertoire, and the multiple sets of *V*, *D*, and *J* gene segments encoding the V region of H chains have their own significance for antibody diversity. In striking contrast, the diversity of the L chain repertoire is very limited. This could be related to an important function the L chain plays to protect from self-reactivity [90]. It has been hypothesized that *H* and *L* chain genes may have co-evolved to ensure that appropriate L chains become available for each H chain that is generated during the early stages of B cell development.

Genes encoding the different L chain isotypes, unlike those of the H chains, are always found at separate loci. Understanding phylogenetic relationships among *IgL* chain genes is difficult since these genes are short and rapidly evolving. However, IgL chains have been found in all vertebrate Ig isotypes, with the exception of camelid HCAbs and shark IgNAR, both lacking L chains, as stated above. Four isotypes have been identified— κ, λ (divided into sub-types in bony fish), σ1, and σ2. All isotypes are present in fish, but the isotype σ2 appears to have been lost in amphibians and σ1 in other tetrapods [91,92], whereas the λ isotype is the only L chain type found in birds [93].

Antibodies are secreted by plasma cells in different degrees of polymerization of the basic unit H_2_L_2_. During evolution, antibodies changed the polymerization degree in their secreted forms—IgM exist as monomers or pentamers in cartilaginous fish; the polymeric form in teleosts is tetrameric, while hexameric in amphibians, pentameric in birds, and pentameric or hexameric in mammals. Different arrangements of the basic unit depend upon the presence of an additional component, termed J peptide, and on the occurrence and the position of the cysteine residues that form inter-subunit disulfide bonds. Avian IgY, and mammalian IgG, and IgE are present only in the monomeric forms.

At this point, it would be interesting to report the current knowledge of the main issues related to the evolution of tetrapod H chain isotypes and sub-isotypes. However, this goes far beyond the aim of the present review.

## 9. Evolution Sites of the Antibody Molecule Involved in Effector Functions

The antibody molecule has been meticulously shaped by evolution in order to enable all its functions. It carries out them in two different steps, which are (i) antigenic recognition, performed by the Fab portions, which comprise the L chains and the two N-terminal domains of the H chain (VH and CH1) and (ii) activation of the cellular mechanisms that neutralize the antigen, conducted by the Fc region, which comprises the constant domains adjacent to the first one. The ability of the antibody molecule to assume different conformations to recognize different partners is ensured by its flexibility. Fab arms bind to antigen through the CDRs of both IgV domains of H and L chain, while the Fc region interacts with Fc receptors, bacterial antigens called “superantigens,” complement factor C1q.

### 9.1. Fc Receptors

The antibody Fc region mediates diverse effector functions by engaging distinct classes of Ig receptors. These receptors are called FcRs because bind to the Fc fragment of antibodies that are attached to a bacterium or to an infected cell. FcRs appeared during the vertebrate evolution and are isotype-specific; they share protein and gene structure, indicating that originated from a common ancestor by gene duplications. [94]. FcRs have not been found in cartilaginous fish but are present in teleosts; IgG and IgE specific receptors appeared during early mammalian evolution [95]. It should be noted that the avian FcR specific for the IgY isotype is the only one that does not comprise an Ig domain in the extracellular portion but presents 8 C-type lectin-like domains [96]. Upon FcR engagement, the antibody molecule triggers the effector cell response that consists essentially in the phagocytosis or cytotoxicity. This antibody activity is primarily dependent on the immune cell types ligated, which express different subsets of Fc receptors. These diversified features contribute to the antibody’s tailored functionality.

The interaction between IgG and Fcγ receptors occurs at the hinge region and the N-terminus of CH2 and is dependent on glycans bound to Asn 297, which stabilize an asymmetrical conformation of Fc; the stoichiometry of the binding is 1:1, differently from Fcα, which binds dimeric IgA [97].

Apart from the classical FcRs, other receptors bind to the antibody Fc region—the polymeric Ig receptor (pIgR) and the so-called neonatal Fc receptor (nFc). pIgRs are integral single-pass transmembrane glycoproteins that facilitate the transcytosis of polymeric immunoglobulins and immunocomplexes [98]. This molecule has two to five extracellular IgV domains, depending on the species, and one non-homologous domain devoted to the proteolytic cleavage of the Ig-pIgR complex. Teleost pIgR exhibits two Ig domains corresponding to II and IV mammalian domains—transports tetrameric IgM [99] and IgT [100].

nFc has a different structure and evolutionary history [101]. It is specific for mammalian IgG. It has an MHC class I-like structure, consisting of the H chain and β_2_ microglobulin. To bind the antibody molecule, it uses a site other than the MHC class I groove that serves as the binding site for the antigen peptides presented by the TCR. The IgG region involved in the binding is at the boundary between the CH2 and CH3 domains; the FcRn region involved in the binding comprises the β_2_ microglobulin, which binds the C-terminus of CH2.

### 9.2. Factor C1q of the Complement System

An important effector mechanism involving antibodies is the activation of the complement cascade through the “classical” (also known as “lytic”) pathway that leads to lysis of the bacterial cell. The first step of the cascade requires the contact between the antibody molecule (IgG or IgM) and the complement factor C1q, which appeared in the evolution in cartilaginous fish in parallel with IgM [102]. An orthologue of the jawed vertebrate C1q is a lamprey lectin binding N-acetylglucosamine. It has been suggested that C1q appeared as a lectin and may have served as a primitive innate immunity recognition molecule in the classical pathway, after the appearance of IgM in cartilaginous fish. The interaction with IgG containing immunocomplexes occurs through the chain B globular end of C1q and the F–G loop of the CH2 domain in IgG or CH3 in IgM [103].

### 9.3. Superantigens

Finally, we should mention the bacterial proteins called “B cell superantigens” or immunoglobulin binding proteins (IBPs), which bind the antibody protein at sites other than that of the recognition of common antigens. Protein A (*Staphylococcus aureus*), Protein G (group C *Streptococci*), the M-protein (group A *Streptococci*) [104], and the Protein L (*Finegoldia magna*) are some of the IBPs studied in more detail. In the case of soluble IgG, the superantigen interaction site is usually at the IgG interface between the second and third constant domains [105], whereas it is located in the V domain of the H chain, in the case of the membrane-bound antibody within the B cell receptor, and does not involve CDRs. It has to be underlined that Protein A interaction is restricted to VH3 family members and Protein G can also bind to the IgG CH1 domain. Protein L binds to the L chain of Igs [106]. The interactions between antibodies and superantigens might be at the basis of a systemic strategy adopted by bacteria to evade the host’s immune response.

## 10. Conclusions

In an attempt to fit together the chapters of the antibody tale, we have provided an overview of the emergence of the structural elements that became, over a long evolutionary time, the most important building blocks to construct the antibody molecule (summarized in Table 1). This scenario recalls the major steps that are those of a general rehearsal, before the advent of the whole molecule with acquired specific functions.

However, only with the emergence of innovative mechanisms generating diversity, antibodies have become a powerful shotgun for the complex adaptive immune system of vertebrates.

Given the vast topic, we have placed a special emphasis on the basic structural features of the antibody molecule, which have made it the main character of the tale, ensuring its evolutionary success. In addition to a certain degree of structural conservation, we have also highlighted some of the most explanatory examples of the ways found by the antibody molecule to reinvent its own canonical structure through unique modifications, while preserving its function.

We know that the picture of the evolutionary origins of the antibody molecule provided in this review is far from over, and we are also aware that it can become clearer as new species in the tree of life will be examined.

## Figures and Tables

**Table 1:** Summary of the emergence in different Phyla of the key elements related to the evolution of antibody. Ig: immunoglobulin; IgV: immunoglobulin variable domain; IgC1: immunoglobulin-constant-region-type 1 domain; RAG: recombination activating genes; AID: activation-induced cytidine deaminase; FcR: crystallizable fragment receptor; pIgR: polymeric Ig receptor. Reference corresponding to each key element is reported in the right-hand column.

Bacteria 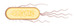	Ig domain	[8]
Porifera 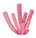	IgV-like domain	[26]
Mollusca 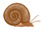	RAG homologs	[44]
Echinodermata 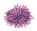	AID homologs	[51]
Protochordates 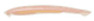	IgV domain	[29]
Agnatha 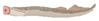	AID	[49]
Chondrichtyes 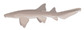	RAG, IgC1 domain, IgNAR, IgM, IgD/IgW	[2]
Osteichtyes 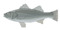	IgT, FcR, RAG, pIgR	[72,95,99]
Amphibia 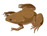	IgX (IgA precursor)	[107]
Aves 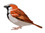	IgY (IgG/IgE precursor)	[85]
Mammals 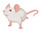	HCAb, IgG, IgE	[69,79]
Homo sapiens 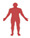	IgG1, IgG2, IgG3, IgG4 subisotype	[108]
